# Breath-by-breath assessment of acute pulmonary edema using electrical impedance tomography, spirometry and volumetric capnography in a sheep (*Ovis Aries*) model

**DOI:** 10.3389/fvets.2024.1402748

**Published:** 2024-07-10

**Authors:** Anthea Raisis, Martina Mosing, Muriel Sacks, Giselle Hosgood, Johannes Schramel, Sarah Blumer, Stephan H. Böhm

**Affiliations:** ^1^School of Veterinary Medicine, Murdoch University, Murdoch, WA, Australia; ^2^Anaesthesiology and Perioperative Intensive Care, Department for Companion Animals and Horses, University of Veterinary Medicine Vienna, Vienna, Austria; ^3^Department of Veterinary Anaesthesia and Analgesia, School of Veterinary Medicine, Murdoch University, Perth, WA, Australia; ^4^Department of Anesthesiology, Intensive Care Medicine and Pain Therapy, Rostock University Medical Centre, Rostock, Germany

**Keywords:** distribution of ventilation, end-expiratory lung impedance, silent spaces, pulmonary function, xylazine, pulmonary edema, electrical impedance tomography

## Abstract

**Background:**

The bedside diagnosis of acute pulmonary edema is challenging. This study evaluated the breath-by-breath information from electrical impedance tomography (EIT), respiratory mechanics and volumetric capnography (VCap) to assess acute pulmonary edema induced by xylazine administration in anesthetized sheep.

**Objective:**

To determine the ability and efficiency of each monitoring modality in detecting changes in lung function associated with onset of pulmonary edema.

**Methods:**

Twenty healthy ewes were anesthetized, positioned in sternal (prone) recumbency and instrumented. Synchronized recordings of EIT, spirometry and VCap were performed for 60 s prior to start of injection, during xylazine injection over 60 s (0–60 s) and continuously for 1 min (60–120 s) after the end of injection. After visual assessment of the recorded mean variables, statistical analysis was performed using a mixed effect model for repeated measures with Bonferroni’s correction for multiple comparisons, to determine at which breath after start of injection the variable was significantly different from baseline. A significant change over time was defined as an adjusted *p* < 0.05. All statistics were performed using GraphPad Prism 0.1.0.

**Results:**

Electrical impedance tomography showed significant changes from baseline in all but two variables. These changes were observed simultaneously during xylazine injection at 48 s and were consistent with development of edema in dependent lung (decreased end-expiratory lung impedance, ventilation in centro-ventral and ventral lung region) and shift of ventilation into non-dependent lung (decreased non-dependent silent spaces and increased center of ventilation ventral to dorsal and increased ventilation in centro-dorsal and dorsal lung region). All changes in lung mechanics also occurred during injection, including decreased dynamic respiratory system compliance and increased peak expiratory flow, peak inspiratory pressure and airway resistance at 48, 54 and 60 s, respectively. Changes in VCap variables were delayed with all occurring after completion of the injection.

**Conclusion:**

In this model of pulmonary edema, EIT detected significant and rapid change in all assessed variables of lung function with changes in regional ventilation indicative of pulmonary edema. Volumetric capnography complemented the EIT findings, while respiratory mechanics were not specific to lung edema. Thus, EIT offers the most comprehensive method for pulmonary edema evaluation, including the assessment of ventilation distribution, thereby enhancing diagnostic capabilities.

## 1 Introduction

Acute onset of noncardiogenic pulmonary edema and associated hypoxemia is the most common cause of respiratory failure requiring mechanical ventilation in critically ill patients with acute respiratory distress syndrome (ARDS). Early recognition of developing pulmonary edema and instigation of appropriate therapy may ultimately allow mortality to be reduced (1).

Spirometry is the “classical” monitoring modality used at the bedside for the assessment of global changes in respiratory mechanics that would be expected with development of pulmonary edema such as reduced compliance, associated with consolidation of dependent lung lobes with fluid, and increased airway resistance, associated with airway narrowing during pulmonary edema (2). Volumetric capnography (VCap) can provide additional information on pulmonary function including indirect information on matching of ventilation and perfusion. Mathematical approximation of the carbon dioxide (CO_2_) curve produced by plotting exhaled CO_2_ tension against the volume of one exhaled tidal breath (3) allows calculation of airway dead space (VDaw), alveolar ventilation (VTalv), Bohr’s physiological dead space (VDBohr), alveolar dead space (VDalv) and the volume of expired CO_2_ per breath (VCO_2_br) (4–6). The sensitivity of this modality to detect early changes associated with developing pulmonary edema is not known. Spirometry and volumetric capnography in critically ill patients require a device to be placed between the breathing system and endotracheal tube (ETT), thus measuring flow and gas composition at the patient’s mouth. Flow and calculated variables can be influenced by diameter and position of the endotracheal tube introducing possible inaccuracies. Furthermore, depending on methods used, measurement of compliance may not differentiate between chest wall or lung compliance. Compliance can also be altered by various lung and airway pathologies resulting in a non-specific measure of respiratory mechanics in disease (7, 8).

Chest radiography and computed tomography (CT) are currently the most common imaging modalities used for diagnosing acute pulmonary edema (9). However, bedside application of CT is impractical, and frequent repeated use of radiography and CT for monitoring progression of pulmonary disease is limited by radiation safety. Electrical impedance tomography (EIT) on the other hand is an emerging non-invasive radiation free real time imaging technology that has the potential to assess pulmonary function, not just globally, but within different regions of the lung (10, 11). EIT simply requires the placement of a belt containing electrodes around the thorax and can be used at the bedside. A weak alternating current is injected by one pair of electrodes at a time and resulting voltages are measured by the remaining electrodes. The process is repeated with injecting current cycles through all electrodes in rapid succession. The measured voltage data is then used to reconstruct cross-sectional images (11, 12). Advantages of EIT include the non-invasive nature of the technology, allowing pulmonary pathology to be assessed continuously without a face mask or ETT, all of which can alter pulmonary mechanics (13, 14). Recently it has been successfully used to identify the changes associated with cardiogenic pulmonary edema in horses (15). As with respiratory mechanics and VCap, the ability of EIT variables to detect early development of edema is not known. However, the ability of continuous non-invasive monitoring provides the potential for detecting regional and global changes on a breath-by-breath basis.

Xylazine administration causes significant changes in pulmonary function in sheep within 2 min of intravenous administration leading to a high prevalence of hypoxemia. The most commonly proposed mechanism for the development of acute pulmonary edema is acute inflammation (16–21). Since the pulmonary edema caused by xylazine are treatable with the alpha2 receptor antagonist atipamezole (22), this is an excellent model to explore the changes in pulmonary function caused by alveolar and interstitial pulmonary edema and bronchoconstriction without having to sacrifice the animals. Furthermore, the xylazine model has the advantage over an oleic acid-induced lung edema of not being influenced by the associated inflammation within the lung tissue (23).

The main objective of this study was to compare the classical bedside monitoring modality spirometry assessing respiratory mechanics with EIT and VCap in their ability to detect acute pulmonary edema. The specific aims were to (i) assess breath-by-breath changes in global variables of pulmonary function measured using EIT, spirometry and VCap; (ii) assess breath by-breath changes in regional variables of pulmonary function measured using EIT and (iii) determine which variables and monitoring modality revealed the first significant change in pulmonary function during the development of acute pulmonary edema. We hypothesized, that (i) all monitoring modalities would be able to detect chances in pulmonary function caused by the development of pulmonary edema, (ii) EIT regional variables would reveal a change in the distribution of ventilation and (iii) EIT would show changes specific to pulmonary edema sooner than the other modalities.

## 2 Materials and methods

The study was approved by the Institution Animal Ethics Committee (R2891/16) in accordance with the Australian code for the care and use of animals for scientific purposes (2013). The sheep used in this study were also included in a study assessing effects of different diets on body composition assessed under anesthesia.

### 2.1 Animals

A total of 20 Merino ewes with a median age of 5 (range 5–6) years were included. Sheep were purchased from breech strike resistant flocks maintained at the Great Southern Agricultural Research Institute and Glenridge, Mount Barker, and transported to the university’s feedlot by researchers in accordance with the code of practice for transportation of sheep in Western Australia (2003). All animals were fully vaccinated and dewormed prior to purchase.

### 2.2 Housing, feeding and enrichment

The ewes were acclimatized for 14 days after arrival to become accustomed to the housing and closer human interactions. Housing consisted of individual pens specifically designed for sheep within a well-ventilated building. The use of pellets with a longer fiber structure, high fiber content, and low protein content was chosen to increase time spent eating and provide enrichment. Enrichment was further provided by housing sheep in individual pens with clear lines of sight of other animals throughout the space, thus simulating a flock environment. All handling was performed by experienced staff using low stress handling techniques. On the day of anesthesia, health was confirmed by inspection, weighing, body condition score and performance of physical examination and analysis of complete blood count.

### 2.3 Anesthesia

For drug administration, a 20 g cannula (Angiocath™ Becton Dickinson, Australia) was placed in the cephalic vein after clipping and sterile preparation of the site. Sheep were sedated with diazepam (Pamlin, Parnell Laboratories, Australia) 0.2 mg/kg administered intravenously (IV). Anesthesia was induced using propofol (Fresefol 1%, Fresensius-Kabi, North Ryde, NSW, Australia) 2–3 mg/kg IV until adequate depth of anesthesia was obtained to allow orotracheal intubation with a 10.5 mm endotracheal tube. Sheep were positioned in sternal recumbency, intubated and the ETT attached to the breathing circuit of the GE Aestiva 5 workstation (Datex Ohmeda, General Electric Healthcare, Australia-New Zealand). Maintenance of anesthesia was achieved using isoflurane (Isothesia® NXR Henry Shein) delivered in oxygen. Standard anesthetic monitoring was performed using the multiparameter monitor Surgivet V9203 (Sound Veterinary Equipment, Rowville, Victoria, Australia) and included heart rate, non-invasive blood pressure and pulse oximetry. Data from the monitor were manually recorded every 5 min. Lungs were mechanically ventilated using a volume-controlled ventilation mode (VCV) with a tidal volume of 10 mL/kg and a respiratory frequency (f*R*) of 10 breaths per minute with zero PEEP throughout the study period. An orogastric tube was inserted into the rumen to prevent gas accumulation.

After completion of the pulmonary function study, instrumentation was removed, and data for the nutritional study was collected. After completion of study, anesthesia was discontinued (25 min after induction of anesthesia) and sheep were moved to individual recovery pens using portable anesthetic trolleys. The sheep were positioned in sternal recumbency using hay bales. Orotracheal intubation with inflated cuff was maintained until sheep were actively swallowing. The cuff was then deflated and ETT removed. The head was elevated to prevent passive regurgitation with nose lower than pharynx to allow saliva to drain. Airway patency and breathing rate and pattern were assessed to ensure return to normal. Sheep were individually monitored until able to stand. After complete recovery, sheep were returned to the animal house, where food and water were provided immediately.

### 2.4 Equipment and data acquisition

EIT data was collected as single plane data set with a custom-made belt, on which 32 electrodes were equidistantly mounted, placed around the thorax, at the level of the 6th intercostal space at mid thorax. Prior to placement, the wool was parted, and non-conductive ultrasound gel was applied to the skin. The belt was then placed over the ultrasound gel and a bandage wrapped over the EIT belt to help hold it in place and to guarantee good electrical skin-electrode contact. The belt was connected to the EIT device BBVet (EIT-branch, SenTec AG, Landquart, Switzerland). Data was recorded continuously at a frame rate of 47 images per second using a customized veterinary software package.

Respiratory mechanics and VCap data were recorded using the NICO capnography device (Respironics, Wallingford, CT, USA), its infrared sensor having a response time of less than 60 ms and an accuracy of ±2 mmHg. Flow, pressure and gasses were measured via a mainstream sensor placed between the ETT and the Y-piece of the breathing circuit. Before each experiment the capnograph was calibrated with room air following the manufacturer’s guidelines while the accuracy of the pneumotachograph of ±5% was confirmed with a 400 mL calibration syringe before and after each experiment. Data were recorded on a breath-by-breath basis using the dedicated software Datacoll (Respironics, Wallingford, CT, USA).

### 2.5 Synchronization and real time data collection

To allow retrospective synchronization of EIT, respiratory mechanics and VCap, mechanical ventilation was stopped for 10 s prior to the start of data collection. Data from each modality were analyzed starting from breath 1 after the restart of mechanical ventilation. To ensure the absence of leakage from the breathing system at increasing airway pressures, inspired and expired VT were compared during each sample time confirming that their difference remained below 10% of the first breath. Data for each breath from 60 s before until the start of the administration of xylazine served as baseline. Subsequently data during xylazine administration (0–60 s) and following xylazine administration (61–360 s) was recorded.

After baseline measurement, xylazine 37.5 μg/kg diluted to 10 mL was administered intravenously over exactly 60 s. To reduce the presence of pulmonary edema and optimize pulmonary function on recovery, all sheep received atipamezole (Antisedan, Pfizer, Sydney Australia) 0.2 mg/kg IM after completion of the entire pulmonary study.

Throughout the data collection period, real time EIT images demonstrating changes in expiratory lung impedance (EELI) were captured for subsequent visual assessment.

### 2.6 Post-hoc analysis

Variables recorded using EIT, respiratory mechanics and VCap are presented below:

#### 2.6.1 Global EIT variables

All impedance values were measured in arbitrary units (AU), which can show marked interindividual variation. Thus, their changes from baseline are expressed in %. The tidal impedance variation (TIV) from the beginning to the end of inspiration was calculated as a surrogate for tidal volume. Impedance at the start of inspiration is referred to as EELI and represents the impedance in the chest before ventilation occurs. It is used as a surrogate for functional residual capacity (FRC).

#### 2.6.2 Regional EIT variables

The center of ventilation expresses the geometric focal point of overall ventilation as a single value (24). The vertical (CoV_VD_) or horizontal (CoV_RL_) position of the CoV is expressed as a percentage of the ventro-dorsal or right–left extension of the lung region, respectively. For CoV_VD_ 0% refers to ventilation occurring in the most ventral (dependent) part of the lung and the very right lung respectively, whereas 100% refers to that in the most dorsal (non-dependent) part and the very left part of the left lung, respectively (11, 25).

Ventilation-induced impedance changes below 10% of the maximum value within the predefined lung area based on a sheep-specific finite element model were determined and these regions defined as ‘Silent Space’. They were divided into dependent (DSS) and non-dependent silent space (NSS) and expressed as a percentage of the entire lung region (25, 26).

To describe the ventro-dorsal distribution of ventilation during inspiration in more detail the entire lung area was divided into four vertical portions of equal width (ventral 25% = ∆Z_V_; centro-ventral 25% = ∆Z_CV_; centro-dorsal 25% = ∆Z_CD_; dorsal 25% = ∆Z_D_). Due to sternal prone position0 of the sheep these regions represent dependent (ventral, centroventral) and non-dependant (centro-dorsal and dorsal) lung regions.

#### 2.6.3 Volumetric capnography

Volumetric capnography variables were calculated by importing the recorded NICO data into a custom-made macro routine in Excel (Excel; Microsoft Corporation, WA, USA) that performs the curve fitting and calculations according to Tusman et al. (27). Airway dead space (VDaw) was measured as the volume from the start of expiration until the inflection point of the VCap curve. Alveolar tidal volume (VTalv) was calculated from the inflection point to the end of the VCap curve. The angle between slope II and III (a-angle), the slope of phase III (SIII), the slope of phase II (SII) the *Y* intercept of the slope of phase III (Yint), *X* intercept of phase II (Xint) carbon dioxide elimination per breath (VCO_2_br) and mixed expired carbon dioxide tension (PĒCO_2_) were also determined from the VCap curve. Bohr’s dead space ratio (VDBohr) was calculated according to the formula given by Tusman et al. (27).

#### 2.6.4 Spirometry variables

Respiratory mechanics were calculated using Flow Tool physiological waveform viewer (flowtool-2910, Respironics Nova Metrix). The rate of air flow was calculated from the pressure difference measured as air moved across the resistance element within the fixed orifice of the pneumotachograph. Expired tidal volume (VT) was calculated as a time integral of the expiratory flow signal. Airway resistance (Raw) was calculated as flow divided by [peak inspiratory pressure (PIP) – plateau pressure]. Dynamic compliance was calculated as VT divided by (PIP-peak expiratory pressure).

#### 2.6.5 Statistics

No power analysis was performed prior to commencing the study as the animal numbers were dictated by the nutritional study. All variables were found to be normally distributed using Shapiro Wilk test and assessment of Q-Q plots, thus parametric statistics were used for all subsequent analysis. The mean of each variable measured in all included sheep was calculated for each synchronized breath and then graphically displayed over time from 60 s prior to, 60 s during and 300 s post-injection of xylazine. Visual assessment of the breath-by-breath changes in these mean variables indicated that most changes already occurred before the end of xylazine administration (60 s) and all obvious changes occurred within 60 s after the end of its injection. Based on these visual findings, all subsequent analyses were performed on data measured from −60 s to 120 s.

The mean of each variable recorded from 60 s before injection to the start of injection was used to establish the baseline for subsequent analysis. For each breath, the mean and 95% confidence interval of each variable was calculated. To allow comparison of the magnitude of change in each variable over time the means recorded at each breath following start of xyalkzine administration were expressed as a percentage of the baseline. The mean of these dependent variables recorded for each mechanical breath was compared to baseline using a mixed effect model. Thus each breath delivered every 6 s (ie time = independent variable) from start of xylazine administration, during the minute of xylazine administration to 1 min after end of xylazine administration was compared to baseline resulting in 23 paired comparisons. Bonferroni test for multiple comparisons was performed with significance defined by an adjusted *p* value of <0.05 (ie *p* < 0.002 × 23). All statistics were performed using GraphPad Prism 0.1.0 (GraphPad Software, LLC, Boston USA).

## 3 Results

Of the 20 ewes, five were excluded from the assessment of pulmonary function due to development of spontaneous breathing within the 120 s after start of xylazine injection. This ‘fighting of the ventilator’ prevented collection of respiratory mechanics and volumetric capnography data.

**Figure 1:**
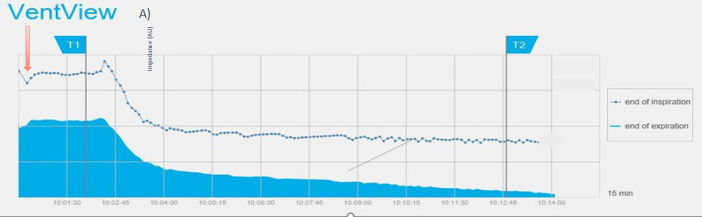
Screenshot of EIT-device showing the trend of end-expiratory (solid blue) and end-inspiratory (blue line) impedance over 15 min. The red arrow indicates the time of breath hold for synchronization recordings, T1 the start of xylazine injection and T2 the administration of atipamezole. The lower gray curve shows five typical volume-controlled breaths.

**Figure 2:**
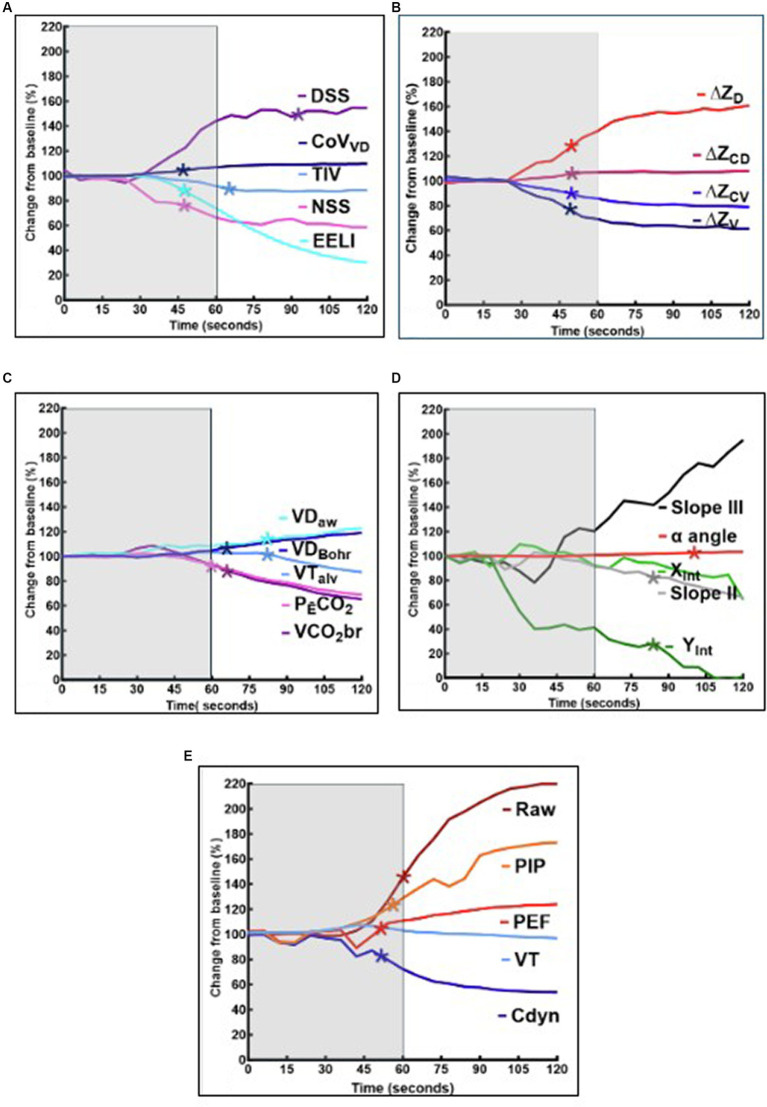
**(A–E)** Graphical representation of the mean variables from 15 sheep, expressed as a percentage of baseline (average of the measurements recorded 60 s prior to xylazine injection). The mean of variables recorded at each breath during xylazine injection and following injection are presented. Significant change from baseline for each specific variable (*p* < 0.05) is represented by an asterix (*). The shaded area represents the 60 s that xylazine is injected. **(A)** Presents EIT variables including tidal impedance variation (TIV), center of ventilation ventral to dorsal (CoV VD), End expiratory lung impedance (EELI), Non-dependent silent space (NSS) and dependent silent spaces (DSS). **(B)** Presents EIT variables including regional ventilation in dependent ie ventral (∆ZV) centro-ventral (∆ZCV) lung regions and non-dependent lung regions ie centro-dorsal (∆ZCD) and dorsal (∆ZD). **(C)** Presents the airway dead space (VDaw) physiological dead space (VDBohr), alveolar ventilation (VTalv) and carbon dioxide elimination (VCO2br; PĒCO2) calculated using volumetric capnography. **(D)** Presents slopes of phase II (SII) and III (SIII), *X* interface of slope II (Xint), *Y* interface of slope III (Yint) and alpha angle of the volume capnograph. **(E)** Presents lung mechanics including dynamic airway compliance (Cdyn), peak expiratory flow (PEF), Peak inspiratory pressure (PIP) and Airway resistance (Raw).

Bedside assessment of EELI revealed consistent marked decreases in EELI commencing during administration of xylazine. An example of the real time changes of EELI is provided in Figure 1. The mean and 95% confidence intervals for each variable of each breath is presented in Table 1 (EIT and respiratory mechanics) and 2 (VCap). The mean % change for variables measured using EIT, respiratory mechanics and VCap over time are displayed in Figures 2A–D.

**Table 1:** (a–d) Mean [95% confidence interval] for variables from 15 sheep recorded for each breath using EIT (a), volumetric capnography (b and c) and respiratory mechanics (d).

(a) Variables derived from EIT										
Time (seconds)		CoV_VD_ %	TIV (AU)	EELI (AU)	NSS %	DSS %	Z_V_ %	Z_CV_ %	Z_CD_ %	Z_D_ (%)
Baseline		52.8 [44.9–59.2]	1,460 [1344–1,576]	2,445 [2068–2,822]	7.04 [4.16–9.92]	8.42 [5.82–11.0]	7.64 [5.90–9.34]	36.9 {33.7–40.1]	41.8 [39.9–43.7]	13.7 [10.7–16.7]
During injection performed over 60 s	0	52.7 [50.3–55.0]	1,470 [1347–1,595]	2,448 [2072–2,825]	7.08 [4.02–10.1]	8.45 [6.10–10.8]	7.87 [6.14–9.60]	37.2 [34.0–40.4]	41.5 [39.6–43.4]	13.4 [10.4–16.5]
	6	52.8 [50.4–55.1]	1,479 [1359–1,599]	2,436 [2062–2,809]	6.97 [4.17–9.78]	8.23 [5.54–10.9]	7.80 [6.06–9.54]	37.0 [33.9–40.2]	41.6 [39.7–43.5]	13.5 [10.6–16.5]
	12	52.8 [50.5–55.2]	1,481 [1361–1,600]	2,439 [2066–2,813]	6.94 [3.99–9.89]	8.31 [5.74–10.9]	7.72 [5.98–9.47]	36.9 [33.8–40.1]	41.8 [39.9–43.7]	13.6 [10.6–16.5]
	18	52.8 [50.5–55.1]	1,485 [1362–1,607]	2,447 [2072–2,823]	6.94 [4.01–9.87]	8.26 [5.65–10.9]	7.75 [6.01–9.49]	37.0 [33.8–40.1]	41.6 [39.7–43.6]	13.6 [10.7–16.5]
	24	52.9 [50.5–55.2]	1,491 [1362–1,620]	2,451 [2061–2,840]	6.97 [3.89–10.1]	8.09 [5.37–10.8]	7.70 [5.91–9.50]	37.0 [33.8–40.1]	41.7 [39.7–43.7]	13.6 [10.7–16.6]
	30	53.7 [51.1–56.2]	1,509 [1363–1,655]	2,444 [2040–2,849]	6.45 [3.34–9.55]	8.37 [5.52–11.2]	7.23 [5.34–9.11]	35.7 [32.3–39.2]	42.4 [40.3–44.5]	14.7 [11.4–17.9]
	36	54.3 [51.8–56.7]	1,478 [1331–1,625]	2,413 [2006–2,819]	5.82 [2.81–8.82]	8.83 [6.04–11.6]	6.82 [5.03–8.60]	34.9 [31.6–38.2]	42.8 [40.8–44.9]	15.4 [12.3–18.5]
	42	54.6 [51.9–57.3]	1,448 [1309–1,586]	2,309 [1916–2,701]	5.82 [2.80–8.83]	9.22 [6.36–12.1]	6.59 [4.82–8.37]	34.5 [30.6–38.4]	43.2 [41.0–45.3]	15.8 [12.2–19.4]
	48	**55.3 *p* = 0.002 [53.8–58.5]**	1,448 [1309–1,586]	**2,149 (0.01) [1769–2,529]**	**5.49 (*p* = 0.03) [2.87–8.11]**	9.79 [2.92–12.7]	**6.03 (*p* = 0.006) [4.47–7.59]**	**33.5 (*p* = 0.002) [29.9–37.1]**	**43.9 (*p* = 0.002) [42.0–45.7]**	**16.6 (0.003) [13.2–20.0]**
	54	**56.2 [53.8–58.3]**	1,415 [1269–1,561]	**1985 [1628–2,342]**	**5.21 [2.55–7.88]**	10.6 [7.85–13.4]	**5.50 [4.00–7.01]**	**32.2 [28.7–35.7]**	**44.5 [42.7–46.4]**	**17.7 [14.5–20.9]**
Post-injection	60	**56.5 [54.0–59.0]**	1,368 [1221–1,513]	**1816 [1478–2,153]**	**4.94 [2.24–7.63]**	11.0 [8.49–13.6]	**5.36 [3.82–6.91]**	**31.7 [28.1–35.4]**	**44.6 [42.7–46.6]**	**18.3 [14.9–21.6]**
	66	**57.0 [54.4–59.7]**	**1,326 (p = 0.03) [1182–1,471]**	**1,637 [1324–1949]**	**4.66 [2.00–7.33]**	11.3 [8.49–13.6]	**5.17 [3.53–6.80]**	**30.9 [27.0–34.8]**	**44.8 [42.6–46.9]**	**19.2 [15.7–22.7]**
	72	**57.3 [54.7–59.8]**	**1,308 [1170–1,447]**	**1,455 [1152–1756]**	**4.39 [2.13–6.65]**	11.2 [8.66–13.7]	**5.10 [3.56–6.65]**	**30.5 [26.7–34.3]**	**44.8 [42.9–46.7]**	**19.6 [16.0–23.2]**
	78	**57.5 [54.9–60.1]**	**1,313 [1173–1,453]**	**1,308 [1026–1,589]**	**4.33 [1.87–6.80]**	11.7 [8.91–14.4]	**4.98 [3.39–6.57]**	**30.1 [26.2–34.0]**	**45.0 [42.9–47.0]**	**20.0 [16.3–23.6]**
	84	**57.7 [55.0–60.4]**	**1,311 [1172–1,450]**	**1,169 [899–1,440]**	**4.64 [2.07–7.20]**	11.6 [8.87–14.3]	**5.01 [3.43–6.59]**	**29.9 [25.8–33.9]**	**44.8 [42.7–46.8]**	**20.4 [16.5–24.1]**
	90	**57.6 [54.9–60.3]**	**1,299 [1170–1,427]**	**1,055 [801–1,309]**	**4.64 [2.19–7.08]**	11.4 [8.49–14.3]	**5.02 [3.44–6.59]**	**30.1 [26.1–34.2]**	**44.6 [42.6–46.6]**	**20.3 [16.5–24.1]**
	96	**57.7 [54.9–60.5]**	**1,305 [1176–1,434]**	**961 [721–1,201]**	**4.42 [1.88–6.96]**	**11.9 (*p* = 0.047) [8.79–14.9]**	**4.97 [3.32–6.62]**	**29.8 [25.7–34.0]**	**44.7 [42.6–46.8]**	**20.5 [16.6–24.3]**
	102	**57.9 [55.2–60.5]**	**1,305 [1178–1,433]**	**877 [649–1,105]**	**4.33 [1.98–6.69]**	**12.0 [8.83–15.1]**	**4.91 [3.37–6.45]**	**29.6 [25.5–33.6]**	**44.8 [42.8–46.8]**	**20.7 [16.9–24.5]**
	108	**57.8 [55.1–60.4]**	**1,298 [1174–1,422]**	**821 [598–1,045]**	**4.22 [1.94–6.51]**	**11.9 [8.80–15.0]**	**4.97 [3.39–6.55]**	**29.6 [25.6–33.6]**	**44.9 [42.8–46.9]**	**20.6 [16.8–24.3]**
	114	**57.9 [55.3–60.6]**	**1,315 [1197–1,434]**	**764 [541–987]**	**4.11 [1.81–6.42]**	**12.3 [9.03–15.5]**	**4.83 [3.27–6.39]**	**29.5 [25.5–33.4]**	**45.0 [43.0–47.0]**	**20.7 [16.9–24.4]**
	120	**58.0 [55.5–60.5]**	**1,309 [1196–1,423]**	**732 [510–954]**	**4.09 [1.80–6.38]**	**12.4 [9.20–15.6]**	**4.81 [3.31–6.31]**	**29.3 [25.5–33.1]**	**45.0 [43.2–46.9]**	**20.8 [17.2–24.5]**

(b) Lung volumes and CO_2_ output measured using volumetric capnography						
Time (seconds)		VTalv (mL)	VDaw (mL)	VDBohr	VCO_2_ br (mL/minute)	PĒCO2 (mL/breath)
Baseline		320 [290−349]	279 [255−303]	0.46 [0.44−0.48]	20.8 [19.3−22.4]	20.8 [19.3−22.4]
During injection performed over 60 seconds	0	324 [292−356]	282 [253−311]	0.47 [0.44−0.49]	20.9 [19.3−22.4]	20.6 [19.2−22.1]
	6	322 [289−355]	286 [261−312]	0.47 [0.44−0.49]	20.8 [19.4−22.2]	20.7 [19.2−22.1]
	12	320 [293−348]	288 [265−310]	0.47 [0.45−0.49]	20.8 [19.4−22.2]	20.6 [19.2−22]
	18	322 [293−352]	288 [263−313]	0.47 [0.44−0.49]	20.7 [19.3−22.1]	20.6 [19.2−21.9]
	24	330 [303−357]	290 [262−318]	0.47 [0.44−0.49]	20.6 [19.3−22.0]	20.7 [19.5−21.9]
	30	348 [317−378]	287 [260−315]	0.46 [0.44−0.48]	20.8 [19.6−22.0]	21.1 [19.8−22.4]
	36	354 [325−383]	305 [278−332]	0.46 [0.44−0.48]	20.1 [18.9−21.3]	20.7 [19−22.4]
	42	336 [297−375]	310 [283−346]	0.47 [0.45−0.5]	19.6 [18.3−20.8]	20.3 [18.3−22.4]
	48	329 [296−361]	298 [265−331]	0.47 [0.44−0.5]	19.8 [18.5−21.0]	20.4 [18.6−22.2]
	54	318 [288−348]	301 [271−331]	0.48 [0.45−0.51]	19.1 [17.8−20.3]	19.7 [18−21.4]
Post-injection	60	313 [283−342]	300 [269−331]	0.48 [0.45−0.51]	18.7 [17.3−20.0]	**19.2 [17.5**−**20.8]**
	66	297 [266−328]	306 [276−336]	**0.5 (*p*=0.03) [0.47−0.52]**	**18.1 (*p*=0.017) [16.7−19.4]**	**18.5 (*p*=0.02) [17−20]**
	72	296 [265−327]	302 [276−329]	**0.5 [0.48−0.52]**	**17.6 [16.1−19.1]**	**17.8 [16.4−19.3]**
	78	284 [251−316]	309 [282−337]	**0.51 [0.49−0.53]**	**17.1 [15.4−18.7]**	**17.1 [15.6−18.6]**
	84	**276 (*p*=0.04) [247−305]**	**316 (*p*=0.03) [292−341]**	**0.52 [0.5−0.53]**	**16.6 [15.0−18.2]**	**16.7 [15.2−18.1]**
	90	**270 [243−297]**	**323 [298−347]**	**0.52 [0.51−0.54]**	**16.3 [14.7−18.0]**	**16.3 [14.8−17.8]**
	96	**267 [242−292]**	**322 [297−347]**	**0.53 [0.51−0.54]**	**15.8 [14.2−17.4]**	**15.8 [14.3−17.3]**
	102	**260 [234−285]**	**326 [304−347]**	**0.53 [0.52−0.55]**	**15.2 [13.8−16.5]**	**15.2 [13.9−16.5]**
	108	**248 [223−273]**	**333 [306−359]**	**0.54 [0.52−0.56]**	**14.8 [13.5−16.2]**	**14.9 [13.6−16.1]**
	114	**250 [220−281]**	**336 [307−366]**	**0.54 [0.52−0.56]**	**14.2 [12.7−15.8]**	**14.3 [13−15.7]**
	120	**245 [214−275]**	**339 [306−372]**	**0.55 [0.53−0.57]**	**14.0 [12.5−15.5]**	**14.1 [12.8−15.5]**

(c) Slopes of volumetric capnograph						
Time (seconds)		Slope of phase III	Intercept of slope III (Yint)	Slope of phase II	Intercept of slope II (Xint)	Alpha angle
Baseline		0.04 [0.03−0.06]	20.6 [16.0−25.2]	0.28 [0.24−0.31]	−54.9 [−61.7−48.1]	167 [164−169]
During injection performed over 60 seconds	0	0.04 [0.03−0.05]	17.1 [10.9−23.3]	0.26 [0.22−0.31]	−55.0 [−61.8−48.2]	168 [166−170]	
	6	0.04 [0.03−0.05]	18.9 [12.5−25.2]	0.26 [0.22−0.3]	−54.6 [−62.1−47.1]	168 [165−170]	
	12	0.04 [0.03−0.05]	19.4 [12.9−25.8]	0.27 [0.23−0.31]	−57.4 [−64.4−50.5]	168 [166−170]	
	18	0.04 [0.03−0.05]	19.5 [14.1−24.9]	0.28 [0.23−0.32]	−59.5 [−67.4−51.5]	167 [165−170]	
	24	0.04 [0.03−0.05]	20.8 [16.6−25.1]	0.25 [0.21−0.3]	−57.0 [−67.8−46.2]	167 [165−170]	
	30	0.04 [0.03−0.05]	21.1 [15.3−26.9]	0.26 [0.22−0.31]	−61.4 [−71.4−51.3]	167 [164−170]	
	36	0.04 [0.03−0.05]	19.7 [13.7−25.7]	0.28 [0.23−0.33]	−64.7 [−72.2−57.2]	166 [164−169]	
	42	0.05 [0.03−0.06]	19.7 [12.5−26.9]	0.28 [0.24−0.33]	−63.2 [−69.8−56.7]	166 [163−169]	
	48	0.05 [0.04−0.06]	19.6 [11.8−27.5]	0.27 [0.22−0.31]	−59.1 [−67.2—51.0]	167 [164−170]	
	54	0.05 [0.04−0.06]	16.9 [10−23.8]	0.26 [0.22−0.3]	−59.2 [−67.0−51.4]	168 [165−170]
Post-injection	60	0.05 [0.04−0.06]	15 [8.45−21.6]	0.25 [0.21−0.29]	−56.8 [−65.0−48.5]	169 [166−171]
	66	0.06 [0.04−0.07]	15.2 [8.86−21.6]	0.24 [0.2−0.29]	−55.6 [−64.8−46.4]	169 [166−171]
	72	0.06 [0.05−0.07]	12 [4.59−19.4]	0.23 [0.18−0.29]	−54.4 [−63.9−44.9]	170 [167−172]
	78	0.06 [0.05−0.07]	9.95 [2.78−17.1]	0.24 [0.19−0.29]	−56.4 [−65.6−47.1]	169 [167−172]
	84	0.06 [0.05−0.07]	**8.4 (*p*=0.02) [2.33−14.5]**	**0.23 (*p*=0.04) [0.18−0.27]**	−53.9 [−62.6−45.2]	170 [168−173]
	90	0.06 [0.05−0.07]	**8.44 [1.88−15]**	**0.22 [0.18−0.27]**	−53.8 [−62.5−45.1]	170 [168−173]
	96	0.07 [0.06−0.08]	**6.18 [−0.36−12.7]**	**0.21 (*p*=0.02) [0.17−0.26]**	−51.9 [−60.7−43.0]	**171 (*p*=0.02) [169−174]**
	102	0.07 [0.05−0.08]	**3.52 [−3.21−10.3]**	**0.21 [0.16−0.25]**	−49.9 [−58.7−41.0]	**172 [170−174]**
	108	0.07 [0.05−0.08]	**2.8 [−2.96−8.56]**	**0.2 [0.15−0.24]**	−48.7 [−58.4−39.0]	**172 [170−174]**
	114	0.06 [0.03−0.09]	**0.95 [−5.64−7.55]**	**0.19 [0.15−0.24]**	−48.6 [−58.8−38.4]	**173 [170−175]**
	120	0	**0.38 [−6.49−7.25]**	**0.18 [0.07−0.30]**	−44.5 [−69.3−19.7]	**173 [170−175]**

(d) Lung mechanics measured using spirometry						
	Time (sec)	VT (mL)	PIP (cmH_2_0)	PEF (mL/minute)	Cdyn (mL/cmH_2_0)	Raw (cmH_2_0/L/second)
Baseline	−60 to−6	594 [559−628]	11.1 [9.90−12.3]	38.6 [36.0−41.1]	90.9 [78.0−104]	3.66 [2.98=4.34]
During injection performed over 60 seconds	0	598 [559−637]	11.1 [9.89−12.4]	39.4 [37.1−41.6]	90.9 [77−105]	3.63 [2.91−4.34]	
	6	599 [559−638]	11.2 [9.91−12.4]	39.6 [37−42.2]	91.4 [77.5−105]	3.61 [2.94−4.28]	
	12	608 [572−643]	11.1 [9.85−12.3]	38.8 [36.5−41.1]	94.1 [81.6−107]	3.54 [2.83−4.25]	
	18	608 [573−643]	11.1 [9.86−12.3]	38.6 [35.6−41.6]	92.9 [79.8−106]	3.66 [2.92−4.4]	
	24	602 [563−641]	11.3 [10.1−12.4]	38.8 [36.2−41.3]	90 [77.4−103]	3.68 [3.02−4.33]	
	30	613 [570−655]	11.4 [10.2−12.5]	39.4 [37−41.7]	87.9 [76.2−99.7]	3.65 [2.98−4.31]	
	36	624 [579−668]	11.6 [10.4−12.8]	39.6 [37.2−42]	86.8 [74.6−99.1]	3.65 [2.99−4.3]	
	42	639 [584−694]	11.6 [10.3−12.9]	39.8 [36.9−42.8]	85.2 [72.1−98.3]	3.68 [3.07−4.29]	
	48	629 [578−680]	12.1 [10.7−13.5]	**40.6 (*p*=0.03) [37.7−43.5]**	**81.4 (*p*=0.002) [68.7−94.1]**	3.88 [3.25−4.5]	
	54	620 [576−665]	**13.3 (*p*=0.001) [11.5−15.1]**	**41.7 [38.9−44.6]**	**73.2 [61−85.3]**	4.6 [3.74−5.46]
Post−injection	60	608 [560−655]	**14.2 [12.1−16.3]**	**42.5 [39.7−45.4]**	**66.6 [54.4−78.8]**	**5.29 (*p*=0.03) [4.16−6.42]**
	66	598 [547−649]	**15.1 [12.8−17.5]**	**43.1 [40.3−46]**	**61.7 [49.6−73.8]**	**5.91 [4.6−7.22]**
	72	596 [544−648]	**15.9 [13.4−18.4]**	**44.2 [41.2−47.2]**	**57.9 [46.4−69.4]**	**6.4 [4.9−7.9]**
	78	589 [533−646]	**16.7 [13.6−19.8]**	**44.3 [41.3−47.2]**	**56.5 [43.9−69.1]**	**7.08 [5.36−8.8]**
	84	589 [536−642]	**17.5 [14−20.9]**	**45.1 [41.7−48.5]**	**54.2 [41.7−66.7]**	**7.3 [5.5−9.1]**
	90	589 [543−635]	**18 [14.6−21.4]**	**46.1 [42.9−49.2]**	**53.6 [42.1−65]**	**7.43 [5.71−9.15]**
	96	585 [541−630]	**18.4 [14.9−22]**	**46.7 [43.4−50]**	**51.8 [41−62.6]**	**7.65 [5.9−9.39]**
	102	579 [536−622]	**18.7 [15−22.4]**	**46.8 [43.4−50.2]**	**51 [40.2−61.9]**	**7.82 [6.04−9.6]**
	108	575 [532−618]	**18.9 [15.1−22.7]**	**47.2 [44−50.4]**	**50.6 [39.8−61.5]**	**7.9 [6.06−9.74]**
	114	573 [530−615]	**19.1 [15.2−23]**	**47.3 [44.1−50.5]**	**50.2 [39.4−61]**	**7.95 [6.15−9.76]**
	120	570 [527−613]	**19.2 [15.3−23.1]**	**47.5 [44.2−50.8]**	**50 [39.4−60.6]**	**7.95 [6.19−9.72]**

Data is presented for each breath recorded at baseline (average of 10 breaths recorded 60 s recorded prior to start of injection), 10 breaths recorded during the 60 s of injection (0–54 s) and 10 breaths recorded 60 s after administration of xylazine (60–120 s). The variables that are significantly different from baseline are shaded in gray. The *p*-value at the time the variable first became significant (*p* < 0.05) is provided. (a) presents the variables measured using EIT, (b,c) presents variables measured using volumetric capnography and (d) presents the variables of lung mechanics. Tidal impedance variation (TIV), center of ventilation ventral to dorsal (CoV VD), End expiratory lung impedance (EELI), Non-dependent silent space (NSS), dependent silent spaces (DSS), regional ventilation ventral (∆Z_V_) centro-ventral (∆Z_CV_), centro-dorsal (∆Z_CD_) and dorsal (∆Z_D_) Lung regions, airway dead space (VDaw) physiological dead space (VDBohr), alveolar ventilation (VTalv)and carbon dioxide elimination per breath (VCO_2_br) Mixed expired carbon dioxide tension (PĒCO_2_), slopes of phase II (SII) and III (SIII), *X* interface of slope II (Xint), *Y* interface of slope III (Yint) and alpha (α) angle, dynamic airway compliance (Cdyn), peak expiratory flow (PEF), pPeak inspiratory pressure (PIP) and airway resistance (Raw). The *p*  value is provided at the time a variable becomes statistically different from baseline. All variables in bold are statistically significant from baseline. The baseline represents the mean and CI of the average of breaths during the minute prior to administration of xylazine. The *p*-value is indicated at the time a specific variable becomes significantly different from baseline. All numbers presented in “Bold” indicate the variable remains significantly different from baseline.

For EIT, significant changes were detected in all standard variables measured. There was a significant decrease in EELI (*p* = 0.01), ∆Z_V_ (*p* = 0.006) ∆Z_CV_ (*p* = 0.002) and increase in CoV_VD_ (*p* = 0.002), ∆Z_CD_ (*p* = 0.002) and ∆Z_D_ (0.003) by 48 s (8 breaths). This was followed by a significant decrease in TIV by 66 s (11 breaths) (*p* = 0.03) after start of injection. Significant increase in NSS (*p* = 0.047), did not occur until 96 s (16 breaths) after start of injection.

For the lung mechanics measured by spirometry, there was a significant decrease in Cdyn (*p* = 0.002) and increase in PEF (*p* = 0.03) by 48 s (8 breaths) after start of injection. This was followed by a significant increase in PIP (*p* = 0.001) and Raw (*p* = 0.03) by 54 s (9 breaths) and 60 s (10 breaths), respectively. There was no significant change detected in VT.

For variables measured using volumetric capnography, significant increase in VDBohr (*p* = 0.05) and significant decreases in PĒCO_2_ (*p* = 0.02) and VCO_2_br (*p* = 0.03) were detected 66 s (11 breaths) after start of injection. This was followed by a significant decrease in VTalv (*p* = 0.04) and significant increase in VDaw (*p* = 0.03) at 84 s (14 breaths). There was also a significant decrease in SIII intercept and S II at 84 s (14 breaths), while alpha angle significantly increased 96 s (16 breaths) (*p* = 0.02) after start of injection.

## 4 Discussion

The study presents the breath-by-breath assessment of EIT variables, volumetric capnography and lung mechanics during development of experimental xylazine-induced acute pulmonary edema. The intent was to identify which of the monitoring modalities would diagnose the pulmonary changes consistent with pulmonary edema in a timely way. Electrical impedance tomography demonstrated rapid and significant changes in all variables with graphical changes evident soon after start of injection. Additionally, EIT provided global and regional variables consistent with fluid accumulation within the lung tissue. Changes in respiratory mechanics, while timely, were inconclusive of either airway or lung tissue changes. Variables calculated from VCap demonstrated delayed changes, with some suggestive of lung tissue changes, but most changes consistent with airway narrowing. EIT and VCap complemented the timely but unspecific findings of respiratory mechanics measurements allowing the early breath-by-breath bedside detection of acute pulmonary edema and associated airway diameter changes.

EIT global and regional variables were the first to show changes specific to fluid accumulation in the lungs (Figure 1A) and statistically significant changes in EELI, CoV_VD_, NSS and regional ventilation during xylazine administration (breath 8 at 48 s). The pronounced decrease in EELI was also evident during real time assessment of EIT recording as demonstrated in Figure 1. These described changes specifically refer to the prone (sternal) position, as observations might vary for animals positioned in dorsal recumbency.

The observed decrease in EELI represents reduced gas within the lung at the end of expiration and therefore decreased FRC. In addition, pneumonia, loss of surfactant and other causes for reduced FRC will decrease EELI (28). Nevertheless, when adding the regional ventilation information and the shift in ventilation toward the non-dependent (dorsal) lung areas to the global EELI variable, rapid fluid accumulation in the dependent (ventral) lung areas can be diagnosed and global changes as for example caused by pneumonia can be ruled out. This shift in ventilation can only be explained by the increase in extravascular lung water in the dependent lung areas in conjunction with gravity. This is best visualized by a spring fixed at the top and the bottom – the ‘Slinky effect’ (15, 29–31). The alveolar edema following xylazine administration collapses the dependent (ventral) areas decreasing the overall ventilated area causing the alveolar matrix to stretch and expand dorsally (32). Graphically this phenomenon became evident within six breaths after start of xylazine injection and was significant two breaths later showing how quickly pulmonary edema occurs – but also how sensitive this EIT variable is to detect fluid accumulation.

The change in respiratory mechanics measured using spirometry, were detected at similar times to changes in EIT variables with decreases in Cdyn and PEF occurring at the same time as changes in EELI and regional ventilation. Interestingly, increases in PIP and Raw occurred after Cdyn and PEF albeit by 1–2 breaths. However, the changes observed, including decreased Cdyn and increased Raw, PIP and PEF are not specific as they are consistent with changes in both lung tissue and airways. As lung edema is associated with changes in airway diameter, respiratory mechanics changes cannot be considered sufficiently specific for bed side monitoring of developing lung edema (2, 19). The use of an inspiratory pause and evaluation of static compliance might have allowed a distinction between airway and parenchymal changes using spirometry (8). In our study we decided to evaluate dynamic compliance as reference variable for respiratory mechanics as this is more widely used clinically.

Compared to EIT, significant changes in VCap variables were delayed compared to other modalities and did not occur until after completion of xylazine administration. The first changes detected by VCap included a decrease in VCO_2_br and P_Ē_CO_2_ and an increase in VD_Bohr_.

The observed decrease in VCO2br can be explained by the pulmonary vasoconstriction described after xylazine administration in sheep (16, 17, 20). Pulmonary vasoconstriction leads to an impairment in gas exchange allowing less CO2 to reach the alveoli.

Another factor decreasing the amount of CO2 exhaled by each breath is increase in physiologic dead space, which we observed in our sheep. However, many of the VCap variables that changed were indicative of airway narrowing including a significant decrease in the intercept of slope III (Yint) and slope of phase II (SII) followed by an increase in alpha angle. It is known that pulmonary edema can cause narrowing of the small airways secondary to pulmonary edema due to loss of radial traction of bronchi as lung volume and FRC decrease (33).

A finding of note when comparing respiratory mechanics and EIT variables is that VT measured via spirometry did not change significantly (Figure 2B). This contrasts with a decrease in TIV evident after completion of xylazine injection (Figure 2A), a variable considered to be a surrogate of VT. The fact that VT did not change was expected as VCV was used. A decrease in mean TIV without a decrease in VT suggests that more gas remained in the large airways and that TIV may be more representative of alveolar ventilation than total ventilation. This is further supported by VCap and the significant decrease in VTalv and increase VDaw (Figure 2C) both of which occurred 2 breaths after the significant decrease in TIV was detected.

An interesting finding was observed when assessing breath-by-breath changes graphically (Figures 2A–D). Between approximately 30 and 48 s (6–8 breaths) following start of injection there is a subtle but consistent “paradoxical” change in many variables for only 2–3 breaths. This includes increased EELI, (Figure 1) VT and Cdyn and a decrease in PEF, VDBohr and VCO2br (Figures 2A–D). These changes were subsequently followed by the marked changes expected in association with development of pulmonary edema. This observation suggests that the lung became temporarily more distensible with a possible widening of the airways at this time. This result was unexpected, and its reason remains unclear.

Despite the appearance of marked changes when visually inspecting the time course of some of the variables, the point in time when statistical significance of such changes was reached was much later that other variables (eg DSS) and for some variables significance was not reached (slope III). This may reflect the relatively small cohort and the individual variation in the response to xylazine. The variance of the parameters for each sequential breath is presented in Table 1.

The delayed significance of the changes of DSS was unexpected considering that other regional variables were clearly indicating a shift of ventilation to non-dependent lung soon after injection of xylazine. As indicated above in addition to the small cohort, this may reflect individual variation in the response to xylazine. Thus statistical significance may not necessarily represent clinical significance. If the early changes in DSS represented varying severity of the disease, DSS could help determine clinical severity. Furthermore, difference between statistical and clinical significance is further exemplified for variables such as CoVVD where statistical significance was detected despite a relatively small % change. In contrast, variables such as EELI, NSS, ∆ZV and ∆ZD demonstrated marked and progressive % changes throughout the measurement period. Further studies are required to determine the relationship between these changes and the clinical relevance.

Other reasons for inability to detect significance in certain variables may represent limitations of the species-specific mathematical finite element model used and the effect of the rumen on the impedance changes observed. This may also be exacerbated by the lens shape of the two-dimensional EIT image which has reduced cranio-caudal spatial resolution (25). In addition, this may reflect lack of sensitivity of certain variables for detecting the changes associated with pulmonary edema. In a recent study investigating congestive heart disease, the authors found novel variables, such as ventilated area, to have greater ability to detect changes associated with congestive heart failure and associated increased extravascular lung water (15). Future studies investigating the use of EIT to assess pulmonary edema should also evaluate novel EIT variables as described in the study by Sacks et al. (15).

The absence of neuromuscular blockers must also be acknowledged as a potential limitation in the study. The presence of increased muscle tone opposing the mechanics of the ventilator may have influenced some results. Breaths including any spontaneous respiratory efforts were excluded by retrospective visual evaluation of flow and pressure curves from every breath included in the analysis.

## 5 Conclusion

This study shows that in a model of acute pulmonary edema created by administration of xylazine to anesthetized sheep, EIT provided useful breath-by-breath information allowing early diagnosis of extravascular lung water. This indicates the use of this technology for clinical assessment of acute pulmonary edema or expected increase in extravascular lung water. The study also showed that more informed interpretation of the changes detected by routinely used spirometry at the bedside was possible with the use of multiple modalities such as EIT and VCap.

## Data availability statement

The original contributions presented in the study are included in the article/supplementary material, further inquiries can be directed to the corresponding author.

## Ethics statement

The animal study was approved by Murdoch University Animal Ethics Committee. The study was conducted in accordance with the local legislation and institutional requirements.

## Author contributions

AR: Conceptualization, Data curation, Investigation, Methodology, Visualization, Writing – original draft, Writing – review & editing. MM: Conceptualization, Data curation, Formal analysis, Investigation, Methodology, Software, Visualization, Writing – original draft, Writing – review & editing. MS: Investigation, Software, Writing – review & editing. GH: Formal analysis, Writing – review & editing. JS: Data curation, Formal analysis, Methodology, Software, Writing – review & editing. SBl: Funding acquisition, Project administration, Resources, Writing – review & editing. SBö: Supervision, Writing – review & editing.
